# *Candida tropicalis* endocarditis: Treatment in a resource-poor setting

**DOI:** 10.4103/0974-2069.74051

**Published:** 2010

**Authors:** Prashant Kumar, Mamta N Muranjan, Milind S Tullu, Pradeep Vaideeswar, Archana Kher, Keya R Lahiri

**Affiliations:** Department of Pediatrics, Seth G.S. Medical College and King Edward Memorial Hospital, Mumbai, India; 1 Department of Pathology (Cardiovascular and Thoracic Division), Seth G.S. Medical College and King Edward Memorial Hospital, Mumbai, India

**Keywords:** Amphotericin B, *Candida tropicalis*, fungal endocarditis, tricuspid valve

## Abstract

Fungal endocarditis (FE) is rare in children and does not usually occur in structurally normal hearts. The commonest causative agent is *Candida albicans*. We report a 5-year-old female child presenting with high-grade fever and cardiac failure. Anemia, leukocytosis and high CRP were found, but bacterial blood culture was sterile. There was no response to antimicrobial agents. Two-dimensional echocardiography revealed a large heterogeneous mass attached to the right ventricle and tricuspid valve. Provisional diagnosis of FE was made, which was confirmed by growth of *Candida tropicalis* in blood culture. Liposomal amphotericin B was started, followed by radical curative surgery including excision of the entire vegetation with total tricuspid valve excision. Histopathology and culture of the resected vegetation confirmed the diagnosis. The patient was given antifungal therapy for a total of 7 weeks, including 2 weeks of post-operative treatment, following which she was afebrile.

## INTRODUCTION

Fungal endocarditis (FE) is uncommon and is associated with a high mortality (56–70%), particularly in developing countries. Two most comprehensive reviews encompassing cases from all age groups reported in English literature from 1965 to 2000 yielded a total of 422 cases.[1,2] Incidence of infective endocarditis in children is 0.8–3.3 per 1000 inpatients.[3,4] The case frequency of FE is about 1.5–4 per 10 million children and it constitutes up to 12% of all pediatric cases of infective endocarditis.[3,4] Among children, 63% of cases are described in neonates and infants younger than 1 year.[3] We report a case of *Candida tropicalis* endocarditis in a child, without any underlying cardiac abnormality or previous cardiac surgery, which fulfilled Duke’s criteria[4] and was confirmed by histopathology and mycology.

## CASE REPORT

A 5-year-old female was admitted with a 7-day history of high-grade fever with chills and dry cough and breathlessness since 4 days. She had been treated (for 40 days) 4 months ago for bilateral empyema (with intercostal drainage and multiple broad spectrum antibiotics- ceftriaxone, cloxacillin and amikacin) and drainage of two cutaneous abscesses, one each in right paravertebral area and on the left forearm. The empyema fluid did not grow any organism. Peripheral blood smear for sickling test was negative. She had required admission to the pediatric intensive care unit (PICU) for mechanical ventilation and central venous pressure monitoring. Enzyme-linked immunosorbent assay (ELISA) for HIV antibodies was negative. At discharge after 46 days of hospital stay, she was asymptomatic but had persistent bilateral pleural thickening with minimal left-sided pleural effusion on the computed tomography (CT) scan.

**Figure 1:**
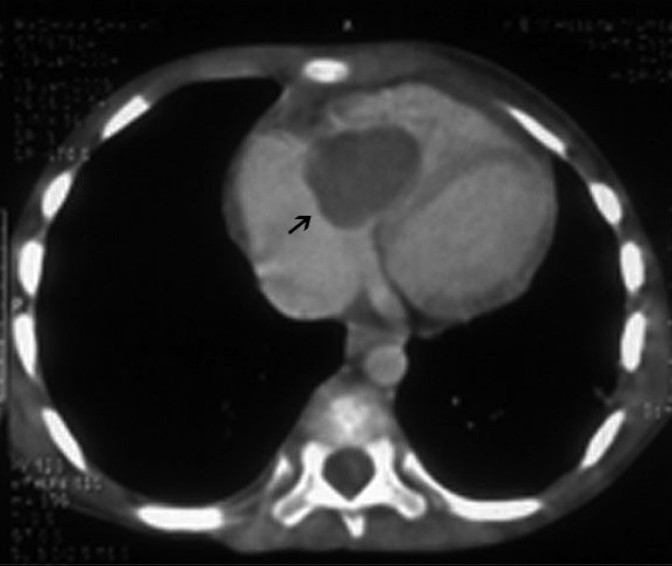
Cardiac CT scan picture showing the vegetation in right ventricle (arrow)

**Figure 2:**
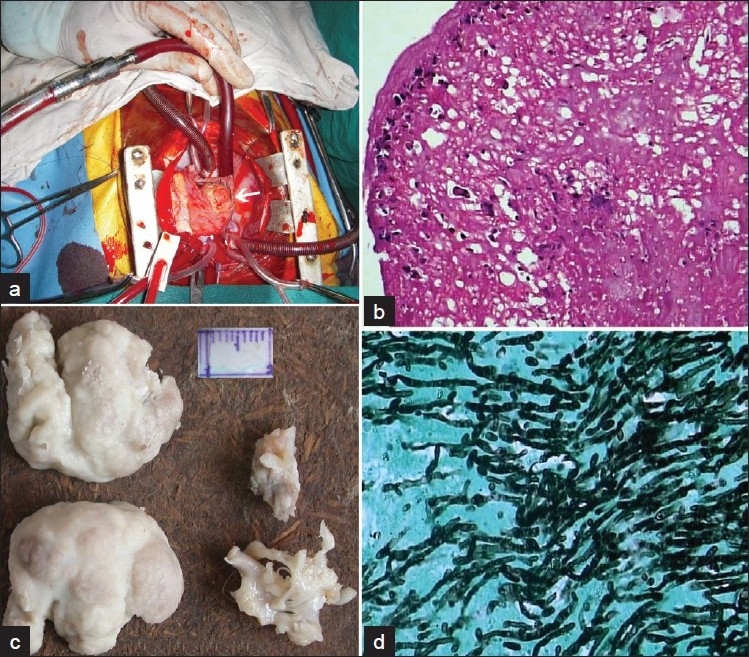
(a) Intraoperative picture of the vegetation (arrow), seen through the right atriotomy; (b) superficial aspect of the vegetation showing neutrophils and clumps of fibrin (H and E, ×400); (c) gross appearance of the resected vegetation and tricuspid valve; (d) yeasts and pseudohyphae, characteristic of *Candida* species (Gomori methenamine silver, ×400)

During the present admission, she had fever with a respiratory rate of 38/minute, intercostal and subcostal retractions, pallor and periorbital edema. Cardiovascular examination revealed a grade 2/6 pansystolic murmur in the tricuspid area, apical pericardial rub and a tender hepatomegaly (liver span of 11 cm). She was malnourished (weight of 14 kg, <5^th^ percentile for age) with a height of 106 cm (25th percentile for age). Investigations revealed hemoglobin 6.5 g/dl, leukocyte count 29,800/mm^3^ (with 81% neutrophils) and platelet count of 71,000/mm^3^. The C - reactive protein (CRP) was 116 mg/l with normal erythrocyte sedimentation rate (ESR) and urinalysis. Blood bacterial culture was sterile. Chest radiograph showed cardiomegaly (cardiothoracic ratio 0.59) without pleural effusion or pleural thickening. The provisional diagnosis was pericardial effusion. Two-dimensional echocardiography showed a 25 × 20 mm heterogeneous mass attached to the base of the right ventricular lateral wall. It prolapsed into the right atrium, producing right ventricular inflow obstruction and grade I tricuspid regurgitation. CT scan of the thorax confirmed the vegetation [Figure 1]. Intravenous ampicillin, cloxacillin and gentamicin were initiated for endocarditis. Furosemide was given for cardiac failure. FE was strongly considered in view of the prior PICU stay, prolonged broad-spectrum antibiotic therapy, indwelling central venous catheter, prolonged chest drainage and presence of large right-sided heterogeneous vegetations. Blood samples were sent for fungal culture and intravenous fluconazole (12 mg/kg loading dose followed by 10 mg/kg/day) was initiated. *C. tropicalis* (species identification done on chrome agar) was cultured on day 7 of admission and fluconazole was substituted by liposomal amphotericin B (3 mg/kg/day, gradually increased to 5 mg/kg/day). Antibiotics were discontinued. As fever and cardiac failure persisted, a repeat echocardiography was done on day 33 of amphotericin B therapy, which revealed an increase in the size of the vegetation to 28 × 28 mm. Therefore, surgical removal of the entire vegetation with total tricuspid valve (TV) excision was performed. Histopathologic examination [Figure 2] and culture of the resected vegetation on Sabouraud’s agar confirmed *C. tropicalis*. Postoperatively, furosemide and digoxin were continued for tricuspid regurgitation to prevent overt cardiac failure. Liposomal amphotericin B was continued for 2 weeks postoperatively (total 7 weeks of therapy). She became afebrile and signs of cardiac failure resolved after 7 days of operation (49 days of admission). The child was discharged after a ward stay of 56 days. Tricuspid valve replacement was envisaged at a later age, as pediatric TV replacement is associated with a poorer prognosis and higher rates of failure than in adults.[5] Moreover, the patient was asymptomatic on digoxin and furosemide despite having free tricuspid regurgitation.

## DISCUSSION

FE most commonly involves the left side of heart (combined aortic and mitral, 70%).[1,2] Tricuspid valve endocarditis (TVE) occurs in 5–10% of cases with infective endocarditis.[6] The usual organisms in TVE are *Staphylococcus aureus* (50–80%) followed by *Pseudomonas aeruginosa* in 10 to 40% of cases. Fungal TVE due to *Candida* species occurs in a mere 3% of cases.[6] The most common risk factors for FE are underlying cardiac disease (congenital or rheumatic), previous cardiac surgery, prosthetic valves, central venous catheters, broad-spectrum antibiotics, immunocompromised states due to immunosuppressant drugs, diabetes mellitus, malignancy or HIV.[1,2] This case had involvement of the native tricuspid valve with no history of prior valve abnormality, which is very uncommon. The most significant risk factor for TVE, which was present in our patient, was prolonged antibiotic use with implanted central venous catheter.[6]

In the present case, the patient was an obviously malnourished child, from a lower socioeconomic class, with illiterate parents. Though poor nutrition may have provided a predisposition to repeated infection and its sequelae (like hospital admission) and endocarditis, it would have been important to rule out an immunodeficiency syndrome. The parents were unable to afford investigations and treatment, and her medical and surgical treatment was entirely funded by our institute. This is a common situation in India where physicians are compelled to use the scant resources for treatment rather than a complete diagnostic work-up.

The commonest causative agent of FE is *Candida albicans*, responsible for 24–46% of all FE.[1–3] Non-albicans species of *Candida* account for a further one-fourth of all cases.[1,2] *Aspergillus* (most commonly *Aspergillus fumigatus*) is the commonest mould causing endocarditis and has been estimated to account for 20–25% of cases in different studies.[1–3] *C. tropicalis* endocarditis is rare in children; only two cases have been reported, a case of sickle-cell anemia with central venous catheter, who developed right atrial thrombus, and subsequently endocarditis which was treated surgically,[7] and a 3-year-old with chronic diarrhea on prolonged total parenteral nutrition was treated successfully with liposomal amphotericin B.[8]

Anecdotal reports document successful medical treatment of FE,[8–11] but most often, as in this case, combined approach with both antifungal therapy and radical surgery offers survival advantage and is recommended.[1,2,12] Amphotericin B is the drug of choice for treatment. The liposomal formulation is preferred because the lesser toxicity permits use of higher, potentially fungicidal doses (up to 5 mg/kg/day) and is often combined with 5-flucytosine (unavailable in India) for synergistic activity. Six to eight weeks of therapy is recommended.[1–3,12] Some experts advise 6 weeks of postoperative amphotericin B to increase the survival, followed by long-term, possibly lifelong fluconazole prophylaxis and follow-up for at least 1 year as the relapse rate is high (30 to 40%).[1,2,12] Fluconazole prophylaxis must be prescribed cautiously in patients with *C. tropicalis* endocarditis as the susceptibility of the species to fluconazole is weak. Additionally, prior fluconazole exposure may encourage infections with fluconazole resistant fungi or candidemia with non-*albicans* species. Due to this and monetary constraints, long-term fluconazole prophylaxis was deferred in our patient.[13]

In a prospective, observational study at 18 medical centers in Italy, including all consecutive patients with a definite diagnosis of IE admitted from January 2004 through December 2007, *Candida* species was the causative organism in eight cases of prosthetic valve endocarditis, five cases of native valve endocarditis, one case of pacemaker endocarditis, and one case of left ventricular patch infection.[14] The *Candida* species accounted for 1.8% of total cases and for 3.4% of prosthetic valve endocarditis cases; the overall mortality rate was 46.6%.[14] Luciani *et al*. have also described a case of a premature infant with mutliple *Candida* tricuspid valve mycetomas wherein eradication of infection was achieved by combined liposomal amphotericin therapy and complex tricuspid valve repair.[15] Similarly, Zenker *et al*. have described three critically ill infants, two weighing less than 1000 g, who survived *Candida* endocarditis without surgery (treatment done with amphotericin B and 5-flucytosine only).[16] The currently described patient was a rare case of tricuspid valve FE due to *C. tropicalis* since he was without any predisposing cardiac abnormality or previous cardiac surgery or immunocompromised state due to drugs, HIV, malignancy or diabetes mellitus, who was successfully treated with a combined medical and surgical approach and the diagnosis confirmed by histopathologic and mycologic methods. The clinicians should think beyond normal barriers in such unusual cases.
